# Many Interesting Items

**Published:** 1895-11

**Authors:** 


### Medical News and Miscellany.

The great scientist has Pasteur better world, let us hope.

---

Dr. Robt. Battey is dead, but his name will live forever in the annals of gynecology.

---

Dr. W. Herring, of San Angelo, county physician of Tom Green county, suicided on October 15th, by taking morphine.

**Dr. W. H. Wathen,** so long the dean of the Kentucky School of Medicine, has resigned, and Dr. Samuel E. Woody has been appointed in his stead.

---

**Important if True.**—It has been ascertained that the potato rot is due to the rot 'tater-y motion of the earth; so says a distinguished common-tater.

---

**Dr. F. E. Hughes,** of Dallas, died October 20, ult., of pneumonia, aged 65 years. Born in Springfield, Ky., August 18, 1830; came to Texas in 1850.

---

**Dr. O. O. Cearsey,** of Temple, Texas, died October 19, ult., aged 80. Dr. Cearsey was a Mexican war veteran. He was not identified with the medical profession of late years.

---

**At the Concert.**—Miss Allie Moad: "Hasn't Mr. Mash a lovely falsetto voice?"

Mr. Strickly Innitt: "Teeth, you mean."

---

**Married.**—At San Antonio, October 2, ult., Dr. A. S. McDaniel to Miss Virgie Gibbs, daughter of Col. C. C. Gibbs, General Land Commissioner of the A. P. R. R., all of San Antonio.

---

**Died.**—At Nacogdoches, Texas, October 2, ult., Mrs. Winfrey Mayfield, wife of Dr. J. E. Mayfield, of that city. The JOURNAL extends its most sincere condolence to the doctor in his great affliction.

---

**At** a recent meeting of the trustees of Jefferson Medical College, Philadelphia, the honorary degree of LL. D. was conferred on Dr. John Collins Warren, Professor of Surgery in Harvard University.

---

**Dr. and Mrs. Wm. A. Adams,** of Fort Worth, have issued invitations to the marriage of their cousin, Miss Margaret Hall Wilson, to Senator Perry Joshua Lewis, of San Antonio, to take place November 5, inst.

---

**Death in the Egg.**—A Mrs. Rorer delivered a lecture on cooking, recently, in New York, and said if a typhoid patient whose temperature has become normal, be given a soft boiled egg, he will suffer a relapse. Nonsense.

**Dr. St. Cloud Cooper** has removed from Jefferson, Texas, to Fort Smith. Arkansas. We are sorry to lose the doctor, and can sincerely congratulate Arkansas upon the acquisition of a valuable addition to the medical guild.

---

**After** pleading and laboring for twenty years with the legislature of Rhode Island, the professsion have succeeded in effecting the passage of a bill which provides for the regulation of medical practice in that State.—*Lancet-Clinic.*

---

**Quarantine Raised.**—Governor Culberson, through his State Health Officer, Swearingen, has issued a proclamation raising the maritime quarantine all along the gulf coast, except as to infected vessels. The station at Galveston will not be closed.

---

**For Sale.**—Our venerable friend Dr. Paulus, recently deceased, left a fine library. Among them is a set of eight volumes of Buck's Reference Hand-Book of Medical Science, as good as new, which will be sold for $35. Address D. A. Paulus, Hallettsville, Texas.

---

**Dr. H. H. Darr,** of Caldwell, Texas, one of the "Red-Back's" staunchest friends, was recently appointed county physician of Burleson county. Dr. Darr takes a deep interest in sanitary matters, and attended the meeting of the American Public Health Association recently.

---

**"Quackery,"** says the *Lancet Clinic*, is the greatest tax borne by the people. It filches the pocket, saps the health, and vitiates the morals of the youth of both sexes. Members of the medical profession are belittled in the eyes of the public by this enemy of mankind, and their influence for good is thereby handicapped.

---

**Dr. B. F. Church,** late First Assistant Superintendent of the North Texas Insane Asylum, at Terrell, having for several years devoted himself to the study of the eye and ear, spent a year in Baltimore recently, perfecting himself in the technique of operations. He has located in Dallas, and has already a fine practice.

---

**Dr. J. F. Hooks,** of Paris, died of blood poison October 19, ult., aged 58. In performing a surgical operation two years ago the doctor inoculated his hand with the pus and has suffered from its effects more or less till it finally caused his death. Dr. Hooks was a leading physician in North Texas and prominent in medical societies.

---

The "Chutmucks" is but another name for an Independent Order of "Good Fellows." As the *New York Medical Record* says, it is "a happy combination of boastful modesty." After they had elected Dr. Love president, they concluded they had better "watch" him, and they did; called "time" on him. He came to time.

---

**Memphis Medical Monthly.**—A monthly Journal of Medicine and Surgery. Established 1880. Subsciption price $1.00 a year. Commencing with the November issue, will publish, each month, liberally illustrated, one or two of the best *clinical* lectures delivered in the Memphis Hospital Medical College. Write for sample copy.

---

**A Remarkable Case.**—Dr. W. R. Blailock, of McGregor, Texas, reports to the JOURNAL an undoubted case of gonorrhœa in a boy of three years, the gonococcus having been demonstrated under the microscope. The history of the case clearly shows it to have been acquired in the usual way; that is, there was "a woman in it," so says the doctor.

---

**Why** should insane people be more troubled with gall stone than other persons? Dr. Taylor says, in his paper in this issue, that 36 per cent. of insane have gall stone; women, of all classes, 25 per cent., and men only 10 per cent. Why? Is there a relation between gall stone and insanity? Certain persons, who are commonly said to have "gall," must be of the 10 per cent. class referred to, eh?

---

**Medical Ethics.**—The *Columbus Medical Journal* says that out West it is not considered ethical for a doctor to have an office in two different villages.

Yes it is, so long as he does not stay at both offices at the same time; that oughtn't to be allowed; it is taking unfair advantage of the other fellows.

---

**Dr. J. J. Wray**, of Dallas, was shot and killed, on the street in Dallas, October 21st ult., by a man named Hardcastle. Wray was unarmed. No cause is known for the killing, but it is said, Hardcastle, whose family physician he was, accused Wray of meddling in his domestic affairs. Both were, we believe, divorced men. Wray was about thirty-six years old, formerly of Mobley, Mo., a graduate of Tulane Medical College, New Orleans.

---

A Physician's Semi-Centennial.—On the 15th of September, 1895, Dr. J. H. Reuss, Sr., of Cuero, celebrated the 50th anniversary of his life as a practicing physician. The doctor is as hale and hearty at the fiftieth mile-post as when he had just begun the journey,—wonderfully preserved in vigor of mind and body. His friends by the hundred flocked to the house to tender congratulations, and to wish many more years of health and happiness. He held a reception all day, and was the recipient of many presents, as well as of congratulations.

---

The Great Pasteur is dead. It seems queer to read of one so distinguished as he,—the foremost, the most famous man in science in France, being admitted to the academy, made a fellow. We know and hear but little of the other fellows into whose society this great man has been admitted as an honor conferred. The boot would appear to fit the other foot best; it would be more appropriate to read of some man's making a discovery, or doing something which rendered him eligible to rank with Pasteur; he gave eclat and distinction to his confreres, and not they to him.

---

We are requested by the Fort Worth Pharmacy Company, of Fort Worth, Texas, to say, that they will be pleased, upon application, to mail their Surgical Instrument Catalogue and Price List to any member of the profession.

Their catalogue quotes over six thousand articles and gives about the same number of cuts or figures of the articles.

They will also be pleased, at request, to mail special catalogues of Electric Batteries, Microscopes, Physicians' Chairs and Tables, and compressed air machines, which are now so successfully used by specialists in the treatment of throat and catarrhal diseases.

---

New Cure for Headache.—An apostle of physical culture says that an excellent and never-failing cure for nervous headache is the simple act of walking backward. Ten minutes is as long as is usually necessary to promenade. It sometimes, however, requires more than ten minutes to walk it off, if one is very "nervous." But it is not understood that it is necessary to walk a chalk-line. Any kind of walking will do, provided it is backward. It is well to get in a long, narrow room, where the windows are high, and walk very slowly, placing first the ball of the foot on the floor, and then the heel. Besides curing the headache, this exercise promotes a graceful carriage. A half hour's walk backward every day will do wonders toward producing a graceful gait.—*N. Y. Med. Record.*

---

**The Great Hot Wells of Corsicana.**—It is not generally known that there are at Corsicana, Navarro county, Texas, on the Central Railroad, the most wonderful wells in the world. The depth is 2477 feet, and the temperature of the water is 126 degrees F. The pressure is 65 pounds,—which will fill stand pipes 150 feet high without pumping, and the output it 300,000 gallons each day. The official analysis shows it to be $99\frac{412}{1000}$ pure, the very small mineral properties contained being absolutely harmless. A Natatorium or palatial bath house is in process of construction which, when completed, will be one of the handsomest and most elegant establishments of its kind in America. In time, it is believed, Corsicana will rival Hot Springs and Carlsbad as a health resort.

---

**Monument to a Martyr Doctor.**—At Montreal recently, a splendid statue in bronze was erected to Dr. Chennier, the Canadian patriot, who, in 1837, led a rebellion against the mother country under circumstances similar to those which drove the American colonies to revolt. He was killed in action. The statue represents him, gun in hand, pointing with his left hand to the enemy, and leading his men. The revolt was quickly put down; but it served to call attention to the wrongs the people complained of,—and for the redress of which they had petitioned Parliament in vain,—and relief quickly followed. Thus good came out of evil, and the Canadians cherish the memory of the doctor somewhat as we do that of Washington. He escaped the stigma which usually attaches to an unsuccessful rebellion; even the government cherishes no resentment towards him.

---

**The New York City Board of Health's New Regulations.**—The New York Board of Health has adopted rules for the prevention of the spread of contagious diseases among public school children. These rules decree that slates and slate pencils shall be abolished, and pens and pencils substituted, the latter to be kept in separate boxes by each pupil. All the school property of a child ill with a contagious disease shall be turned over to the health board. Books that are taken home shall be re-covered with brown paper once a month. Each class must have its own covered pitcher for drinking water, and each pupil a separate cup. In cases of contagious disease in a family, the children are at once to be excluded, and teachers are to report weekly to the board the names and conditions of pupils who are ill. All teachers and principals shall be forbidden to send any pupil to the home of another pupil for any reason. Circulars of instruction to the Board of Education on those matters, and also to principals, are in the course of preparation.

---

**A New Remedy for Boils and Carbuncles, Camphorated Salol.**—It is prepared by moistening one part of camphor with a few drops of alcohol, and rubbing this in a porcelain mortar, with one-fourth part of salol, until a transparent liquid is obtained. "A change takes place," says Dr. Bowen (*Boston Medical and Surgical Journal*), "in from twelve to twenty-four hours; pain diminishes, redness and inflammation of adjoining parts disappear, and the tumor becomes progressively smaller, without the formation of pus. Or, after suppuration has already taken place and after the slough has been removed, the pain and hyperæmia may be much lessened by the application of camphorated salol, and the suppuration diminished. The healing process then advances quickly, a slight discoloration and some infiltration being felt only for a short time. * * * Lay bare the point of the furuncle (or, if carbuncle, make several moderately deep incisions in order to facilitate penetration into the infiltration), cover with cotton compresses soaked in the camphorated salol, and an impermeable covering outside."

---

**Filtration of Public Water Supplies.**—The *New York Medical Journal*, in reviewing Mr. Allen Hazen's recent book on this subject, says: "This book should be in the possession of such boards of public works as are considering the improvement of their interior water supply, especially where the records of recent European and American researches into the subject of filtration on a large scale can not be studied at first hand. Mr. Hazen's connection as chemist at the Laurence station of the Massachusetts State Board of Health, and his familiarity with the great quantity of information incorporated in the last six issues of the reports of that board, together with the fact that he has investigated various European plants which serve as models of filtration on the large scale, all contribute to the merit of the book. * * * * Exhaustive experiments made at Providence, R. I., served to convert the city engineer and some others to the view that there are economic conditions under which mechanical filters are preferable to gravity filters for purifying the water supplied for the use of a city.'' What's the matter with Austin's sending for Hazen for help in her dilemma?

---

**Faith Healer Extraordinary.**—The *Illustrated American* gives an account, accompanied with a picture, of a remarkable person, by name Francis Schlatter, who is imitating Christ in his manners and habits, and who is said to heal, by a pressure of his hands, all manner of lame, halt, sick, deaf and blind. He was formerly a shoemaker at Denver. He says he received a ''command to go forth and heal all who will have faith." He fasted forty days and forty nights and went to healing. He goes barehead and barefoot and dresses in coarse clothes. The most remarkable part of it is that he bears a striking resemblance to the picture usually accepted as that of Jesus. He has mild blue eyes, soft flowing beard and long hair which curls slightly at the end, and he parts it in the middle. There is no doubt that this resemblance and the imitation of the Savior in habits and dress heighten the effect of the mental impression and contribute most to the ''cures.'' He will not enter any person's house (except that of a Mr. Fox whose protége he seems to be) and will take no money or other reward. The *Illustrated American* says he has made cures that are truly marvelous and that *thousands* daily press to see him and be healed. Well—what are we coming to?

---

**Antitoxin in Diphtheria.**—In the *Johns-Hopkins Hospital Bulletin* for July and August, 1895, Dr. Welch has an exhaustive paper on this subject. It is one of the ablest and most important contributions to the literature of antitoxin treatment that has appeared. The *Journal of the American Medical Association* reviews the paper and summarizes the author's conclusion as follows: The results of the treatment of over 7000 cases of diphtheria by antitoxin demonstrates beyond all reasonable doubt that anti-diphtheritic serum is a specific curative agent for diphtheria, surpassing in its efficacy all older methods. It is therefore the positive duty of physicians to use it. * * * The discovery of the healing serum is entirely the result of laboratory work; it is the outcome of the studies of immunity, and was in no sense, accidental." "It should be forcibly brought home to those whose philozooic sentiments outweigh sentiments of true philanthropy, that these discoveries which have led to the saving of untold thousands of human lives have been gained by the sacrifice of the lives of thousands of animals, and by no possibility could have been made without experimentation upon animals."

---

**The Disinfection of Rooms after Infectious Diseases.**—The absolute disinfection and sterilization of a room in which a patient has been treated, is considered at present extremely difficult, if not impossible. We refer, of course, to rooms in ordinary houses which can not be washed down with large amounts of strong antiseptic solutions. The usual procedure of burning quantities of sulphur, of washing the walls and removing all movable pieces of furniture, is considered to be all that is practically possible. Dr. G. Barnet has been working in co-operation with Dr. Trillat, for the purpose of determining whether a better method can not be found for the disinfection of infected rooms, and he claims to have obtained successful results. The substance which he used is a solution of formaldehyde in alcohol. He has devised an apparatus by which the vapors of this substance are diffused through a room, and he says that with it he can disinfect with absolute certainty in six hours three hundred cubic metres of air. Cultures of various specific micro-organisms placed in the room are rendered absolutely sterile by this process. The vapors of this formaldehyde have no injurious action upon the furniture of the room, and they disappear rapidly after a few hours of airing. The process suggested and the apparatus devised appear to us to be rather expensive, but if they do all that Dr. Barnet claims, they will prove a very useful addition to our methods of fighting disease.—*N. Y. Med. Record.*

---

**Then and Now.**—A valued correspondent writes: "Dr. Paulus was one of the best advisors I ever knew. He was capable of concentrating his mind on the subject in hand to the exclusion of all else. In these days of *money*, *money*, *money*, it seems almost absurd to speak in praise of one, who, during so long a professional life as Dr. Panlus, failed to accumulate wealth. Yet he was to those who knew him, the ideal physician, and his life and character and his course present a strong contrast to the "up to date'' doctor; the new set, I mean. Many of them seem to think of nothing but surgery, in its most limited sense (and money getting), and with them their highest ambition is to *cut, cut, cut.* But when thrown with men like Cupples, Paulus and others I could name; or pick up such old books, as *Dixon's Practice*, *Wood's Practice*, Astley Cooper on *Fractures*, or, especially Oliver Wendell Holmes' prize paper on the *Contagiousness of Puerperal Fever* (written, I think, in 1842-3) I feel like exclaiming, 'There were giants in those days!' It was not,—'well, I made sixty-five calls yesterday and expect to make seventy today' that made those men great, but the closest study of each individual case, and the cultivated faculties of observation and deduction; the diagnostic power.''

[Our correspondent is bitter, and a little unjust. There are giants in *these* days, and many of our young doctors are most worthy sons of honored sires—and will be in time, ornaments to the medical profession.]

---

**A Celebrated New Yorker Dead.**—William Henry Schieffelin died at his home recently. He was head of the firm of Schieffelin & Co., wholesale druggists. Mr. Schieffelin was born in this city August 20, 1836. Though educated for a business life his career was checkered with adventure. In 1860 he was chosen to lead an exploration party in the far west. The party crossed the Rocky mountains from Montana on the same trail followed by the Lewis and Clark expeditions, and was captured by the Crow Indians. When on the point of losing their lives they were released by a chief. In 1862, he entered the war of the rebellion in the Seventh regiment of New York. He afterward left the regiment and was commissioned major of the First New York Mounted Rifles, and completed the regiment by enlisting 400 men. In 1863 he resigned his commission and returned to New York in time to take part in suppressing the draft riots. In the same year he married Mary Jay, grand-daughter of Chief Justice John Jay, and devoted himself to the interests of his father's business, which was established in 1794 by Jacob Schieffelin. The firm has now become widely known for its enterprise in introducing synthetic drugs of modern chemistry to American physicians. Mr. Schieffelin was a vestryman in St. George's

Episcopal church, at Stuyvesant square. He was fond of farming, boating and shooting, and was one of the first to import Jersey cattle to this country. Though taking a great interest in public affairs, he was never a candidate for a political office. He was president of the Fisher's Island Sportsman's club, a member of the Union League club since the war, a charter member of the City club and the South Side club, and belonged to the Loyal Legion. Mrs. Schieffelin and two children, William Jay Schieffelin and Eleanor J. Schieffelin survive Mr. Schieffelin.—*N. Y. Times.*

---

**A. P. H. A.**—At the recent meeting of the American Public Health Association at Denver (Oct. 1–4, ult.), seventy new members were admitted. The subject of water pollution and purification of water supplies was extensively discussed, but nothing was done, unless, we except that Dr. McCormack, of Kentucky, introduced a motion which prevailed, to appoint a committee of three, with Dr. Peter H. Bryce, of Toronto (who read a paper on the subject), as chairman, to "formulate a plan, and suggest to congress that something in the way of national legislation in this matter should be done." That has been done time and again, and nothing came of it.

**The New Germicide, "Formalin."**—In the discussion, which followed the reading of the report of the committee on Car Sanitation, by Granville P. Conn, of Concord, Dr. Kinyon, of the M. H. S., stated that "a 15 per cent. volume of burning sulphur is not sufficient to disinfect a sleeping car. Steam will, in a certain sense, prove adequate when used by an experienced or competent person; but an ordinary car can be disinfected in ten minutes by the use of a spray of formalin, which he had found to be the best disinfectant or agent to kill small-pox and other germs. It is more effective than tricresol, or carbolic acid, and is easy to handle." * * * Dr. Conn closed by saying that "formalin is a well known agent, and when it dries, it forms a dust or powder that will kill germs in the carpets and in the other furnishings of our cars."

---

**"Mandrakes."**—A minister friend of the "Red Back," a truly pious and good man, yet one who believes that a Christian has better cause to be cheerful and happy than any other person, and who, consequently, enjoys a joke or a little pleasantry as well as any other fellow, assures the JOURNAL of his keen appreciation of Dr. Merriman's remarks on mandrakes (in our August number); and for Dr. Merriman's information, and to relieve his anxiety, as well as to enlighten our readers who may have become interested in the subject, he sends us the following, which he has dug out of Wordsworth and other commentators:

"Genesis 30:15: '*Give me, I pray thee, of thy son's mandrakes.*' Mandrakes were believed to produce fruitfulness. The Hebrew word is '*dudain*,' supposed to be derived from the root '*dud*,' to love, and substantive, '*dod*,' love; and therefore it is rendered 'love-apples,' and they were used as a philtre to conciliate the affections. By the Arabs, they were called 'Apples of Satan,' and they would seem to correspond with 'Circeta' of the Romans. The Septuagint renders the word 'Mandragoras' [Greek characters omitted], and the Vulgate by 'Mandragoras.' It is the *atropia mandragora* (of Linneus), and resembles the belladonna, with a root like that of the carrot, having white and reddish blossoms, and yellow odoriferous apples which ripen in May and June, have an obnoxious odor, and, when eaten, produce a kind of intoxication. When Leah's four sons were born in succession, she ascribed all these births to the Lord Jehovah; but now we hear an ascription of a birth to Fortune, and she regards it as conducive to her own happiness. Leah did not think of God in connection with these two births; they were only, in her eyes, the successful and welcome result of the means which she herself had used"—[of which there is scarcely room for doubt; regarding the mandrakes *solely* as the "means"; nevertheless, it will be remembered, she borrowed Jacob, we are led to suppose, only to emphasize and give effect to the "means"; to make it "take," as it were.--ED.]

---

**Membrane of the Hen's Egg in Grafting.**—The New York *Medical Journal* gives a very interesting instance of the successful employment of this substance in place of skin-grafting by M. Amat. This surgeon was called to treat a large burn in a boy upon the dorsal region. Repair being very slow the surgeon suggested skin-grafting, but, as this measure was objected to by the parents of the child, the idea came to him that the internal vascular layer of the membrane of the hen's egg might answer the purpose. Accordingly the membrane was employed with satisfactory results. Since this time (some five years since) M.

Amat and others have employed this method of grafting with success in numerous cases.

The technique of this operation is as follows: (1) The membrane should be taken from a very fresh egg, as physiological observation has shown that the latent life of this membrane is then more active. (2) Grafting must not be done until the dressings have suppressed the suppuration and provoked a healthy growth. Previously to the transplantation contact with the air must be avoided by a thick dressing of gauze saturated with a carbolic acid solution. (3) Take a very fresh egg, break it in the center, empty it of the contents, and seize the membrane with a mouse-tooth forceps at the large end of the egg, that is, the internal layer of the membrane of the shell. (4) This layer is cut into strips about four or five millimeters in width and of the same length. These are applied on the wound with the point of a pair of scissors, and laid on their albuminous surface. (5) They are applied at a distance from twelve to fifteen millimeters, and are covered with a small square of tin foil, and then by a dressing of gauze saturated with a solution of carbolic acid. this heteroplastic procedure, says the writer, is worthy of attention, especially from practitioners who do not always have at hand the proper material for "inter-human," or inter-zoohuman" heteroplasty.

Says the *Practitioner and News:* "It may be that the writer intends to teach that the embryonic connective tissue cell may be converted into an epithelium cell by the action of this membrane, but this is at variance with all previous physiological teaching, and if true would quite revolutionize all received doctrines of development.

It may be that the membrane, like the sponge in sponge-grafting, acts simply by giving support to the minute vessels which are concerned in building up the granulating surface of the wound; but while such a process would facilitate the filling up of gaps, etc., it is not easy to see how it could give any desirable covering to a skin-denuded surface of any considerable area.

If egg-membrane grafting is to be put forward as a substitute for skin-grafting, its advocates should surely give us some clear statement of the *methodus medendi* of the new procedure, and a name for the anatomical elements produced by it."

Suppose we call the *modus*, "chicanery" and the operation itself "hen-er-oplastic;" or is the whole *thing an "eggsaggeration?"*

**Honor to whom Honor is Due.**—The origin of the Texas quarantine system. Dr. J. T. Fry, one of the Texas pioneer physicians, in a letter to the *Galveston News*, in response to a request for his autobiography, gives an outline of his life and work; and as it touches upon a subject which for some time has been in controversy, we extract and present herewith the following pointed remarks. Dr. Fry was born in Tennessee, studied medicine, graduated at New Orleans, and came to Texas in 1853, as a young doctor, but did not begin practice till 1856. He saw Galveston grow up from a village.

With reference to the yellow fever, and the necessity for quarantine, he says:

It was in 1853 that the gulf coast was visited and ravaged by the most destructive epidemic of yellow fever known, and it was in this and subsequent years that the writer began to study, and formulated ideas of that scourge which enabled him, in after years, to perform for his adopted State the only work which he claims to have been of any material beneft to it. A pretty close observer in his profession, he became satisfied that the unsettled position of public and medical opinion on the origin and transmission of yellow fever had to be determined. There were strong believers that the disease was exotic, and could be excluded. There were just as firm believers that quarantine was useless, and a stumbling block to travel and commerce; that the disease could and did originate right here, or any favorable place, like New Orleans, and other points along the gulf. Up to 1878, the question was an unadjusted one in the public and medical minds. Fortunately for this writer, he was brought into an epidemic in 1862, in the town of Matagorda, which enabled him to prove decided views. All the circumstances attending it came under his personal observation. With fixed views, he was elected a member of the 16th Legislature, which was in 1878-9, and then it was, under a force of circumstances, such as shotgun quarantine, hesitating ideas and wavering actions, without the guiding powers of effective statutes, he formulated the ideas and drafted them into the bill which became the quarantine law of the State of Texas. Many bills and plans had been offered, discussed, and rejected by the committee on public health and vital statistics, and the whole matter seemed about to be left in its previously chaotic and inefficient state. My bill was drafted after a night's reflection, and was offered in the house, referred and accepted without the alteration of an item or the slightest opposition. I had such a bulwark of support in such men as Colonel (Dr.) Ashbel Smith, Guy M. Bryan, George P. Finley, Senator Benevides, and others, in the house, that the bill went quickly through, and, my fellow citizens, let me indulge the vanity of saying to you for the first time, that under the efficient enforcement of this law by good State health officers, Texas has been rid of this blaster of its progress and rapid growth, the dread destroyer of happy homes and loved ones, for just one-quarter of a century. Imagine for one moment what would have been the condition had yellow fever made its visits as often as it had done previously; but not only this, the result in Texas of establishing a successful quarantine has had its effect on the success of positive and complete quarantine rigidly enforced throughout the United States, if not the world, leading indirectly, if not directly, to the development of successful investigation of bacteria and microbic diseases. This one good I claim to have done for my State, and it is enough. Minor matters shall not be alluded to. A perfect quarantine, if such can be, will forever exclude these scourges of the human race from our homes.

The credit for originating the quarantine idea,—the germ from which, under the enlightened administration of State Health Officer Swearingen, has been evolved and perfected our present rational and effective system,—has heretofore been given to and claimed by Dr. R. Rutherford, who was one year State Health Officer (1882) under Gov. Roberts, and four years under Gov. Ross (1886-90); and Gov. Ross, in his last message, states that Dr. Rutherford was the author of the first bill. The above should forever put the question at rest, and we put it on record in the interest of truth and of history.

---

**Home for Epileptics.**—Apropos of the letter published elsewhere, proposing that Texas shall establish a colony of epileptics after the manner of the one in New York, and as giving valuable information on the subject, we reproduce the following from the *New York Medical Record* of October 12th, ult.:

Craig Colony, named for the late Oscar Craig, of Rochester, formerly President of the State Board of Charities, consists of nearly one thousand nine hundred acres of land in the Genesee Valley. It is reached by two trunk lines of railway (the Erie and the Delaware and Lackawanna) and from roads centering at Rochester by the Western New York & Pennsylvania Railroad. The colony has its own postoffice and railway station, known as Sonyea, an Indian word signifying sunny place. The law establishing the colony required that it should be arranged on the village plan. Craig Colony will not resemble an institution in any particular, but will look more like a country town than anything else. As the patients are received they will be set to work or at study in various ways. They will take care of the farms, gardens and orchards; they will plan and build new houses. There will be among them tailors, shoemakers, printers, bookbinders, masons, ironworkers, carpenters, painters, and so on. In fact, every sort of employment, every sort of recreation, everything in short that goes to make up the life of a country village, will be found in this colony, the only difference being that the citizens of this community will be epileptics. The resources of the land are such that almost everything in the way of food for the inhabitants of the village can be raised by themselves, and their surplus agricultural and manufacturing products, judiciously managed, can make the colony practically self-supporting. Work has been progressing very rapidly during the year to prepare existing buildings for the reception of patients. The first quota of patients, numbering sixty, will be taken from the almshouse early in November. The patients taken from the almshouses and asylums will be known as State patients, and they will be provided for before any private patients can be received. They will be sent to the colony by the poor authorities of each county, according to a form required by law, the blanks for which will be furnished on application to the State Board of Charities, or the superintendent of the colony. As soon as all epileptics now upon public charge eligible for admission to the colony are provided for, private patients will be received at prices to be regulated by the Board of Managers, according to the kind and extent of care and attention required. Such patients may, if it be desired, erect cottages for their own use upon the grounds, upon application to the Board of Managers. There will be no restriction as to the age of patients admitted, and the only restriction practically applies to the mental condition. Insane epileptics, or epileptics subject to insane outbreaks, can not be taken into the colony. The law permits the Board of Managers to take and hold in trust for the State any grant of land, gift, or bequest of money, or any donation to be applied, principal or income, or both, to the maintenance and education of epileptics, and the general uses of the colony. Dr. Frederick Peterson is President of the Board of Managers, and Mr. H. E. Brown, Secretary. The medical superintendent is Dr. William P. Spratling, Craig Colony, Sonyea, N. Y.

---

**Isopathy in Africa.**—Doctor Thirk, in the year 1846, published in the *Medical Weekly*, of Vienna, a very interesting account of so-called "poison physicians" among the Caffirs and Hottentots at the Cape of Good Hope, Africa.

These medicine men claim to cure cases of poisoning which have resulted from snake bites, or from the wounds of poisoned arrows. To enable him to properly prepare himself as a qualified poison physician the following procedure is adopted: He secretes under the article of fur, which constitutes his only clothing, a poisonous scorpion, to whose stings he freely exposes himself. After the reaction resulting from the first sting is accomplished, another sting is accepted, and when the effect is over a third and a fourth, and so on until the body becomes perfectly insensible to the stings of a single scorpion; then he exposes himself to the stings of two in the same manner, then three, and more scorpions, until at last the body seems utterly unaffected by such poison. Advancing further in his preparation, the poison doctor hardens his body in like manner against the bites of a peculiar webless spider which lives in holes; then in like manner against the bites of the crown serpent. And lastly, to complete the charm or invulnerability against poison, he submits to the bites of the puff-adder.

All these preliminaries having been faithfully carried out, the poison doctor is ready to begin the exercise of his art. From time to time, however, he must renew the strength of his healing properties and sustain his reputation as a poison doctor by re-exposing himself to these bites.

The treatment of patients placed under his professional care is affected in the following manner: A piece of the fur cape of the poison doctor, which has been soaked with the medical man's sweat, is then put into some water which the patient is directed to drink. In cases where the poisoning took place some considerable time before applying to the doctor some very offensive doses are swallowed by the patient.

The poisoning of arrows is affected with the secretion from the water of the spider mentioned above with the venom of the crown-snake and the puff-adder mixed with gall. These cases are interesting as illustrations of a savage instinct which recognizes the power of animal extracts as means, not only of inducing serious injury, but as methods to prepare the body to resist these same obnoxious influences. In England we had Jenner's method in vaccination; in Berlin, the tuberculin of Koch; in Paris, the hydrophobin of Pasteur. The subcutaneous injections of Brown-Sequard are in the same line of thought and experiment. It is the evolution of preventive medicine originating in the mind of the untutored savage, and passing onward and upward, until the very highest bacteriological skill confirms its theories for the protection and health of mankind. That the use of animal extracts may be made a potent instrument for good there can be longer any great doubt. Researches of our ablest scientists have, we think, successfully proved this, and discoveries in this line receive the unqualified approval of some of the most distinguished members of the medical profession.—*W. Thornton Parker, M. D., in Popular Science News.*

**A Remedy for Tape-worm.**—Dr. J. H. Newington, writing to *The Lancet*, says that many years ago he was giving a patient a mixture as follows: Hydriodate of potass., gr. xxxvj.; iodine, gr. xij.; water, ℥j.—ten drops three times a day in water. The patient unexpectedly passed a tape-worm eleven yards long, dead, of which there were no previous symptoms. He has since given the same medicine successfully in two or three cases. The last patient came to him about three years since and stated that he had suffered from tape-worm for two years and was constantly passing pieces of the parasite, but could not get rid of it. Dr. Newington gave him the same medicine, and after a short time passed a mass of tape-worm, dead, and there has been no return.

---

### THE JOURNAL'S OWN.

**Wanted**—a young doctor, who is seeking a location, to travel over the State and take subscriptions for the Red Back. It takes like hot cakes, and as a very liberal arrangement will be made with the proper party, it can be made to pay well, and afford an opportunity to select an advantageous location. References imperatively required.

---

**Wanted**—The address of the following Texas physicians: The JOURNAL, sent to their respective addresses as given, has been returned—Dr. B. D. Holbert, formerly of Palestine; Dr. J. A. Jones, of Utica; Dr. J. A. Dobbings, Demings Bridge; Dr. W. T. DeTar, Taylor; Dr. H. W. Dudley, Hot Springs, Ark.; Dr. B. A. Harris, Harold.

---

**Ought to Read the Red-back.**—The *Journal of the American Medical Association* says, "Addresses wanted: Dr. F. Meyer, journal re turned from Baltimore; Dr. M. Perl, journal returned from Houston." Dr. M. Perl is dead, long since; notice published in the TEXAS MEDICAL JOURNAL at the time. Readers of the Red-back will find the news affecting Texas doctors, in each issue.

---

**The "Red-Back"—A Good Suggestion.**—A valued subscriber, in remitting, says: "I being busily engaged with my practice, my wife usually receives my mail. She is a constant reader of the 'Red-Back,' and a few days since, upon my return from a visit, she said: 'Have you sent the "Red-Back" any money lately? A receipt came this morning.' She handed me your 'little reminder,' and I said, 'behold, it is not signed,' and she saw the point. Enclosed find money order. How would it do to send bills to the delinquent's wives? May be the delinquent is busy; ha, ha!"

---

DOLIBER-GOODALE COMPANY,
BOSTON, MASS., October 19, 1895.

*Drs. Daniel & Hudson, Texas Medical Journal, Austin, Texas:*

GENTLEMEN:—We can look back, we assure you, with great pleasure to the many years of pleasant business relations we have had with the TEXAS MEDICAL JOURNAL and its editors, and we have been very much gratified at the many courtesies you have extended to us directly and to our representatives when in Texas during these years. Very truly yours,

DOLIBER-GOODALE CO.

---

"I must confess that I can not very well do without the Red-Back; I prize it more highly than any of the journals I am taking, and I subscribe to seven.

W. M. POWELL, M. D.,
Albany, Texas."

"Enclosed please find a remittance of $2 for renewal of my subscription to your valuable and interesting journal.

R. C. NETTLES, M. D.,
Marlin, Texas."

(☞ For the *eleventh year*.—ED.)

"Instead of showing signs of the decrepitude of age, the Red-Back gets redder, brighter and better all the time.

JARRETTE D. LAW, M. D.,
Salado, Texas."

---

A VALUED SUBSCRIBER in remitting, says: "When you know a man to be good pay, why don't you send a bill for renewal of subscription immediately on expiration of time paid?"

Well, there are many men of many minds—all, more or less, "good pay," who differ very much in their way of thinking. Subscription is payable in advance. We have tried our friend's plan, and have found to our sorrow that some whose patronage and good will we valued highly actually took offense. Some will say, "as I am paid up to date, stop the JOURNAL." We have had many to say, "when I get behind, draw on me." Others, two and three years behind, good men, who have simply neglected to remit, have gotten furiously mad when presented with a draft. That is most remarkable. We have had this thing to occur recently. It would seem to us that to a man who wants to pay and intends to pay for his JOURNAL, but who has simply neglected it, a draft on him would be a convenience, relieving him of writing a letter and getting a money order. The bank man hands him his receipt and he hands the man the money. During stringent times for two years or more, we have let up on collections; have not annoyed our subscribers with bills. But now that prosperity has retured; now that cotton is being marketed; the time come when the doctor collects, if ever, we have sent bills to all our subscribers. After waiting twenty days in a certain per cent. of cases where the party is two or three years in arrears—and has not responded we have made draft. While many have paid, a few have gotten angry; construed it into "an insult," a "doubt of their intention," and *stopped* the JOURNAL. Now, this is very hard and very unjust. We want some one of this class to say what he would do in a case like this; here is a reputable prosperous doctor who has been getting the JOURNAL regularly three or four years since his first paid up subscription expired, who will pay no attention to a bill and will not answer a letter. What course is left us? I see none but to list such a man as a *dead-beat* and charge the amount to profit and loss. Are any of you gentlemen who read this in this category?

